# Corticotropin-releasing factor-like diuretic hormone acts as a gonad-inhibiting hormone in adult female, *Rhodnius prolixus*

**DOI:** 10.3389/fendo.2023.1279929

**Published:** 2023-09-29

**Authors:** Areej N. Al-Dailami, Ian Orchard, Angela B. Lange

**Affiliations:** Department of Biology, University of Toronto Mississauga, Mississauga, ON, Canada

**Keywords:** reproduction, ovary, egg production, egg-laying, hatching, triatomine, kissing bug

## Abstract

Within insects, corticotropin-releasing factor/diuretic hormones (CRF/DHs) are responsible for the modulation of a range of physiological and behavioural processes such as feeding, diuresis, and reproduction. Rhopr-CRF/DH plays a key role in feeding and diuresis in *Rhodnius prolixus*, a blood-gorging insect and a vector for human Chagas disease. Here, we extend our understanding on the role of this neurohormone in reproduction in adult female *R. prolixus*. Double-label immunohistochemistry displays co-localized staining of CRF-like and the glycoprotein hormone (GPA2/GPB5) subunit GPB5-like immunoreactivity in the same neurosecretory cells (NSCs) in the mesothoracic ganglionic mass (MTGM) and in their neurohemal sites in adult female *R. prolixus*, suggesting these peptides could work together to regulate physiological processes. qPCR analysis reveals that the transcript for Rhopr-CRF/DH receptor 2 (*Rhopr-CRF/DH-R2*) is expressed in reproductive tissues and fat body (FB) in adult female *R. prolixus*, and its expression increases post blood meal (PBM), a stimulus that triggers diuresis and reproduction. Using RNA interference, transcript expression of *Rhopr-CRF/DH-R2* was knocked down, and egg production monitored by examining the major yolk protein, vitellogenin (Vg), the number and quality of eggs laid, and their hatching ratio. Injection of dsCRFR2 into adult females reduces *Rhopr-CRF/DH-R2* transcript expression, accelerates oogenesis, increases the number of eggs produced, and reduces hatching rate in female *R. prolixus*. Downregulation of *Rhopr-CRF/DH-R2* leads to an increase in the transcript expression of *RhoprVg1* in the fat body and ovaries, and increases the transcript level for the Vg receptor, *RhoprVgR*, in the ovaries. A significant increase in Vg content in the fat body and in the hemolymph is also observed. Incubation of isolated tissues with Rhopr-CRF/DH leads to a significant decrease in transcript expression of *RhoprVg1* in the fat body and *RhoprVg1* in the ovaries. In addition, Rhopr-CRF/DH reduces transcript expression of the ecdysteroid biosynthetic enzymes and reduces ecdysteroid titer in the culture medium containing isolated ovaries. These results suggest the involvement of the CRF-signaling pathway in reproduction, and that Rhopr-CRF/DH acts as a gonad-inhibiting hormone in the adult female *R. prolixus*, as previously shown for the colocalized glycoprotein, GPA2/GPB5.

## 1 Introduction

In vertebrates, corticotropin-releasing factor (CRF) is a neurohormone modulating various physiological processes in response to stressors, including feeding and reproduction (1). In insects, CRF can also act as a diuretic hormone, and so this family of peptides in insects is referred to as CRF/DHs [see reference (2)]. The first insect CRF/DH was isolated and sequenced in *Manduca sexta* (3), where diuretic activity was shown *in vivo*. Since then, other CRF/DHs have been identified and characterized as diuretic hormones in many insects, including *Locusta migratoria* (4), *Periplaneta americana* (5), *Drosophila melanogaster* (6), *Tenebrio molitor* (7), *Rhodnius prolixus* (8) and *Schistocerca gregaria* (9). The first insect CRF/DH shown to be a true diuretic hormone was Locmi-CRF/DH in *L. migratoria*, where the peptide is present in neurosecretory cells (NSCs) and their neurohemal structures and released into the hemolymph after feeding. Locmi-CRF/DH was shown to stimulate diuresis *in vivo* and to stimulate secretion by Malpighian tubules *in vitro* (10–12).

Along with diuretic activity, CRF/DHs are also implicated in regulating pre-ecdysis behavior, oviduct muscle contractions, dorsal vessel contractions, and female reproduction (see reference 13–15). In the locust, *S. gregaria*, injection of CRF/DH into the hemolymph prior to feeding results in insects consuming a smaller meal (9), thereby suggesting that Schgr-CRF/DH, released at feeding, induces satiation. Also, injection of Schgr-CRF/DH into mature *S. gregaria* females results in the production of significantly smaller oocytes, and reduced ecdysteroid levels circulating in the hemolymph (9). RNA interference (RNAi) knockdown of the transcript for Schgr-CRF/DH results in the opposite effects. The authors concluded that CRF/DH may be part of an integrating control system following a meal, that influences food intake and diuresis, as well as anabolic processes such as oocyte growth and ecdysteroidogenesis.

*Rhodnius prolixus*, a pioneer model organism in the study of insect physiology, is a blood-feeding hemipteran native to Central and South America. Substantial quantities of blood are necessary for high energy demanding activities and have epidemiological relevance such as reproduction, seeking food sources, and dispersion (16). All post-embryonic stages gorge on a blood meal and immediately on gorging, *R. prolixus* triggers diuresis and associated downstream physiological events to remove excess water and salt (17–19). In *R. prolixus*, Rhopr-CRF/DH is a true diuretic hormone (see reference 13), released during blood gorging from lateral NSCs of the mesothoracic ganglionic mass (MTGM), and working synergistically with serotonin to orchestrate post-prandial diuresis (8, 20). As a neurohormone, Rhopr-CRF/DH can have widespread targets, in addition to those related to diuresis. Thus, the transcript for a Rhopr-CRF/DH receptor, *Rhopr-CRF/DH-R2*, is expressed in many tissues in *R. prolixus*, with pronounced expression observed in the testes and ovaries (13). In adults, a significant increase in transcript expression of *Rhopr-CRF/DH-R2* is observed in the adult female ovaries compared to the fifth instar, suggesting that the Rhopr-CRF/DH-signaling pathway has increased activity during egg production. Interestingly, injecting adult females with Rhopr-CRF/DH reduces the number of eggs made and laid suggesting Rhopr-CRF/DH also inhibits reproduction (14). The glycoprotein hormone GPA2/GPB5 was recently reported to participate in regulating reproduction by acting as a gonad-inhibiting hormone in *R. prolixus* (21, 22). It is worth noting the previous observation that GPB5-like immunoreactivity in the MTGM shows a similar neuronal distribution pattern to CRF-like immunoreactivity, especially in the lateral NSCs and their neurohemal sites on the abdominal nerves (21). This suggests that these two neurohormones could be co-localized and released together to regulate reproduction in *R. prolixus*, lending more support to a role for Rhopr-CRF/DH in reproduction.

The aim of this study is to further investigate the role of the Rhopr-CRF/DH-signaling pathway in regulating egg production in adult female *R. prolixus.*

## 2 Materials and methods

### 2.1 Animal

All experiments were performed on adult females taken from an established colony at the University of Toronto Mississauga, maintained in an incubator at 25°C and 50% humidity. The insects were fed on defibrinated rabbit blood (Cedarlane Laboratories Inc., Burlington, ON, Canada) through an artificial feeding membrane once in each instar (20). All insects had a similar feeding and body weight history.

### 2.2 Whole mount immunohistochemistry

Central nervous systems (CNSs) of unfed adult females were dissected in *R, prolixus* physiological saline (150 mM NaCl, 8.6 mM KCl, 2 mM CaCl_2_, 4 mM NaHCO_3_, 34 mM glucose, 8.5 mM MgCl_2_, 5 mM HEPES, pH 7) at room temperature. Fixation and staining were performed as previously described (21, 22), with some modification for the double-labeling as follows; the first primary antiserum used was anti-CRF antiserum at 1:5000 (10), followed by the secondary antiserum Alexa 594 donkey anti-rabbit. Tissues were then washed, and the same procedure repeated with a primary anti-GPB5 antiserum at 1:5000 (21), followed by a secondary antiserum Alexa 488 goat anti-rabbit. Alexa 488 and Alexa 594 have minimal spectral overlap and therefore minimal bleed-through in double-labeling. Images were taken from the dorsal of the CNS and are shown as z projections of 5 stacked images. To check the specificity of the anti-CRF antiserum and the anti-GPB5 antiserum, tissues were stained with preabsorbed CRF antiserum (using 10^−5^ M Rhopr-CRF/DH) or preabsorbed GPB5 antiserum (using 10^−5^ M GPB5 antigen). These controls showed no staining, confirming the specificity of the antisera.

Images were acquired using a confocal microscope LSM-800 (Carl Zeiss, Jena, Germany) and then processed with the Zeiss LSM Image Browser software. Confocal images were prepared using ImageJ Software (https://imagej.nih.gov/ij/).

### 2.3 RNA extraction and reverse transcription quantitative PCR

Total RNA was extracted from tissues of adult female *R. prolixus* using TRIzol reagent (Invitrogen by Thermo Fisher Scientific, MA, USA) according to the manufacturer’s instructions. cDNA samples were synthesized using the High-Capacity cDNA Reverse Transcription Kit (Applied Biosystems, Mississauga, ON, Canada), using 1 µg of total RNA, random primers and 50 U of MultiScribe MuLV reverse transcriptase. qPCR assays were performed as previously described (22). Actin and Rp49 (60S ribosomal protein) housekeeping genes were used as reference genes to normalize the target gene expression. To assess the accuracy of the cDNA product amplification, the dissociation curves were examined and found to have a single peak for each pair of primers (**Supplementary Table 1**). Tissue distribution mRNA expression was calculated relative to 1000 copies of the average of the reference genes using the 2^-ΔCt^ method (23). For the rest of the experiments, the results are shown as fold change quantified relative to the expression of the control samples, using the 2^−ΔΔCt^ method (24). All samples had 4-5 biological replicates with each containing 2 technical replicates and using no-template controls.

### 2.4 Double-stranded RNA synthesis and delivery

To synthesize double stranded RNA (dsRNA), two non-overlapping fragments of *Rhopr-CRF/DH-R2* were prepared by PCR by conjugating the T7 RNA polymerase promoter (5′-taatacgactcactatagggaga-3′) to the 5′ end of the gene specific primers (**Supplementary Table 1**). dsRNA synthesis was performed as described previously (22).

For RNA interference experiments, a group of adult females 10 days post ecdysis (PE) were fed and allowed to copulate for two days prior to injection with dsRNA. Insects were injected with 2 µl of dsCRFR2 (containing 2.5 µg of each dsRNA) into the thoracic/abdominal hemocoel at the base of the metathoracic legs using a 10 μL Hamilton micro syringe (Hamilton Company, NV, USA). A second group of insects was injected with ds ampicillin-related gene (dsARG) as control. All insects were left for 1 h at room temperature and then placed into an incubator at 28°C with 12:12 light/dark cycle. Using qPCR, knockdown of the *Rhopr-CRF/DH-R2* transcript was measured on 2, 5 and 14 days post-injection to determine knockdown efficiency (**Supplementary Figure 1**).

### 2.5 Egg production, laying and hatching

Virgin adult females were individually weighed to record pre-fed weight, then fed through a membrane on defibrinated rabbit blood (Cedarlane Laboratories Inc., Burlington, ON, Canada) for 20 minutes. Immediately after feeding, insects were weighed to verify that they took similar amounts of a blood meal. Only insects who took over 2.5 times their initial body weight were used. Each female was then placed in a cubicle containing two fed adult males and allowed to copulate for two days prior to injection with dsRNA. Copulation was confirmed by the presence of an ejected spermatophore. Adult females were then injected with 2μL of 2.5 µg/µL dsCRFR2 or 2μL of 2 μg/μL dsARG, as described above. At 4 days post-injection (6 days PBM), the fat body, ovaries, and hemolymph were collected and processed for RT-qPCR, enzyme-linked immunosorbent assay (ELISA), Western blot, or protein determination, as indicated. Effects of dsRNA was examined, including the ovarian and egg morphology (photographed with a Leica DVM6 digital microscope (Leica Microsystems, Wetzlar, Germany)), number of eggs laid and hatching ratio. Measurements of egg length and width were recorded and analyzed using ImageJ software to determine differences between treatment groups, and the volume determined using the equation of an ellipsoid = $\frac{\text{π}}{6}$ (width)^2^ (length), given the circular nature of the longitudinal axis (25).

### 2.6 Sample collection and total protein and vitellogenin quantification

Hemolymph, fat body and ovaries were collected from dsARG-injected and dsCRFR2-injected adult females 6 days PBM for total protein and vitellogenin (Vg) measurements.

For hemolymph collection, insects were immobilized on surgical wax and 10 uL hemolymph collected using a Hamilton syringe from cut legs while gently pressing the abdomen. The hemolymph was placed in ice-cold microtubes and then diluted in cold anticoagulant solution (10 mM Na_2_EDTA, 100 mM glucose, 62 mM NaCl, 30 mM sodium citrate, 26 mM citric acid, pH 4.6) at a ratio of 1:5 (anticoagulant: hemolymph) (26). Samples were then centrifuged at 10,000 × g for 10 min at 4°C and the supernatants used for determination of Vg concentration.

Following hemolymph collection, fat bodies and ovaries were dissected under cold *R. prolixus* saline and protein extraction performed with TRIzol reagent according to the manufacturer’s recommendations. Following protein extraction, quantification of total protein was performed using the BCA protein quantification assay (Pierce™ BCA Protein Assay Kit, Thermo Fisher, ON, Canada).

Vitellogenin quantification from the hemolymph, fat bodies, and ovaries was performed using an ELISA as previously described (27). Microtiter plates were loaded with 100 μL/well of standard Vg or with hemolymph, fat bodies and ovaries diluted in carbonate buffer (15 mM Na_2_CO_3_, 35 mM NaHCO_3_, pH 9.6) and incubated for 90 min at 37°C. Following incubation, plates were washed two times with phosphate buffered saline-Tween (PBST: 8.2 mM Na_2_HPO_4_, 1.5 mM KH_2_PO_4_, 150 mM NaCl, 2.7 mM KCl, 0.05% Tween 20, pH 7.4) on a shaker at room temperature for 5 minutes for each wash. Plates were loaded with 100 µL of primary affinity purified rabbit IgG Vg antibody with a concentration of (0.01 µg/mL) prepared in PBST containing 0.1% bovine serum albumin (BSA) for 60 minutes at 37°C. The polyclonal Vg antibody was obtained from Boster Biological Technology (Pleasanton, CA, USA) as previously described (22). Plates were then washed two times as described above followed by loading plates with 100 µL of anti-rabbit immunoglobulin conjugated to horseradish peroxidase (HRP) in PBST (1:4000) for 30 minutes at 37°C. After washing, plates were incubated with 100 µL of the enzyme 3,3′,5,5′-Tetramethylbenzidine (TMB) Liquid Substrate System (Millipore-Sigma, Oakville, ON, Canada) for 15 minutes and then the reaction was stopped by adding 100µL of 1M H_2_SO_4_. Plates were read at 410 nm using a multi-mode reader (Synergy HTX, CA USA).

### 2.7 SDS-PAGE and Western blot

For SDS-PAGE Coomassie staining, pre-made gel (percentage 4 – 20%, Mini-Protean TGX Stain-Free Precast Gels, BioRad, Mississauga, ON, Canada) was used to separate proteins from 1 μL hemolymph of dsRNA-treated insects under reducing conditions, following the manufacturer’s instructions. After electrophoresis, the gel was stained with QC Colloidal Coomassie (BioRad), for 1 h at room temperature with gentle shaking. The gel was then de-stained overnight at 4°C with gentle agitation to remove the background using a de-staining solution of 50% (v/v) methanol in water with 10% (v/v) acetic acid. The gel was imaged on a ChemiDoc XRS system (BioRad). For western blot, 1 μL of hemolymph (1:20 dilution) for each dsRNA treatment was used to separate protein under reducing conditions on pre-made gels, following the method of Leyria et al. (28). Primary anti-Vg antibody at 1:2000 dilution was incubated overnight at 4°C with gentle agitation. For the secondary antibody, HRP-conjugated goat anti-rabbit IgG was used at a dilution of 1:5000 and the gel incubated for 1 h at room temperature with gentle shaking. Blots were visualized using enhanced chemiluminescence (Clarity Western ECL Substrate, BioRad), imaged on a ChemiDoc XRS system and analyzed using Image Lab 5.0 (BioRad Software and System).

### 2.8 *In vitro* experiment: Rhopr-CRF/DH incubation

Isolated fat bodies and ovaries from fed, mated adult females 6 d PBM were incubated individually with Rhopr-CRF/DH (Genscript, Piscataway, NJ, USA) in 300 μL of Grace’s medium (Millipore-Sigma, Oakville, ON, Canada) in 1.5 mL Eppendorf tubes. Rhopr-CRF/DH (3 uL of 10^-6^M) was added to the incubation medium to get a final concentration of 10^-8^ M; for controls 3 uL of saline was added to the incubation medium. The tissues and incubation media were collected after 4 h of incubation (in the dark, at 28°C with gentle shaking) and processed for RT-qPCR, Vg (as described above) and ecdysteroid quantification (see below).

### 2.9 *In vivo* experiment: Rhopr-CRF/DH injection

Fed and mated adult females were injected with either 2 μL of 10^-3^ M Rhopr-CRF/DH or 2 μL of saline at 6 d PBM. The fat bodies and ovaries were dissected 24 h later and processed for RT-qPCR (as described above).

### 2.10 Ecdysteroid quantification by ELISA

Hemolymph from Rhopr-CRF/DH-injected insects or incubation medium from *in vitro* assays were collected to measure ecdysteroid titers. 5 μl of hemolymph was extracted from each insect for each biological sample. Hemolymph was added to methanol at a ratio of 1:3 (hemolymph: methanol), then centrifuged for 10 min at 20,000 g at 4°C to remove precipitated proteins. The supernatants were dried under vacuum in a Vacufuge plus centrifuge (Eppendorf, USA) at room temperature and dissolved in 150 μl of assay buffer containing 0.1% BSA as previously described (29, 30). Competitive ELISA, using 20 hydroxyecdysone (20E)-conjugated to HRP and a rabbit anti-ecdysteroid primary antibody (obtained from Dr. Timothy Kingan), was used to quantify ecdysteroid titers as previously described (29, 30).

### 2.11 Statistical analyses

All graphs are created using the GraphPad Prism Software (GraphPad Software, CA, USA, www.graphpad.com). Significance of differences were determined either with Student’s t-test, with one-way ANOVA followed by Tukey’s *post hoc* test, as indicated.

## 3 Results

### 3.1 CRF-like and GPB5-like immunoreactivity in the MTGM

**Figure 1:**
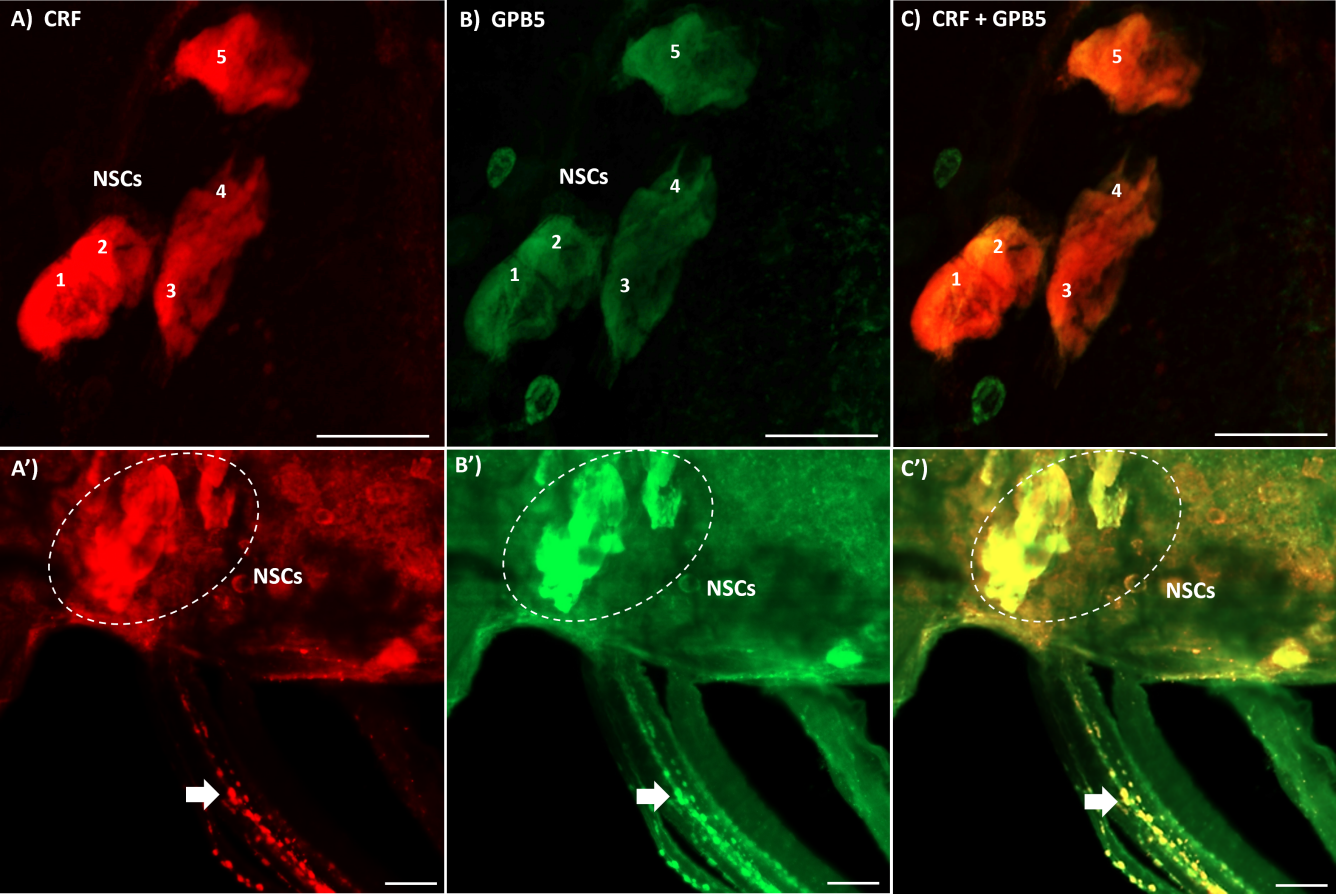
Double-label immunohistochemistry of the mesothoracic ganglionic mass (MTGM) of adult female *R. prolixus.* **(A–C)** show the dorsal view of the MTGM with lateral neurosecretory cells (NSCs) numbered 1-5 stained for corticotropin-releasing factor (CRF)-like immunoreactivity (red), GPB5-like immunoreactivity (green) and a merged image. **(A’–C’)** show the lateral dorsal view of the MTGM with stained NSCs (outlined in white) and double-labeled neurohemal processes on the abdominal nerves (arrows). Scale bars; 50 μM.

Immunoreactivity of CRF-like cells in the CNS of fifth instar and adult *R. prolixus* was previously reported (14, 21). Intense staining of lateral NSCs in the MTGM with processes extending to neurohemal sites located on the abdominal nerves are particularly evident. Interestingly, we recently reported GPB5-like immunoreactivity, showing a similar pattern of staining of lateral NSCs and their neurohemal sites (21). Here, we show that indeed, CRF-like and GPB5-like immunoreactivity is co-localized in the five large lateral NSCs in the MTGM (**Figures 1A–C**) and in their neurohemal processes on the abdominal nerves 1, 2 and 3 (**Figures 1A’–C’**). Note that not all processes and cells in the MTGM show co-localization of CRF-like and GPB5-like immunoreactivity. For controls, the antigens (Rhopr-CRF/DH or GPB5 at 10^-5^ M) were preabsorbed overnight with the antibody at 4°C prior to use (**Supplementary Figure 2**). No staining was found in these controls indicating that the staining was specific to these antigens.

### 3.2 Tissue distribution of *Rhopr-CRF/DH-R2* transcripts in adult females

**Figure 2:**
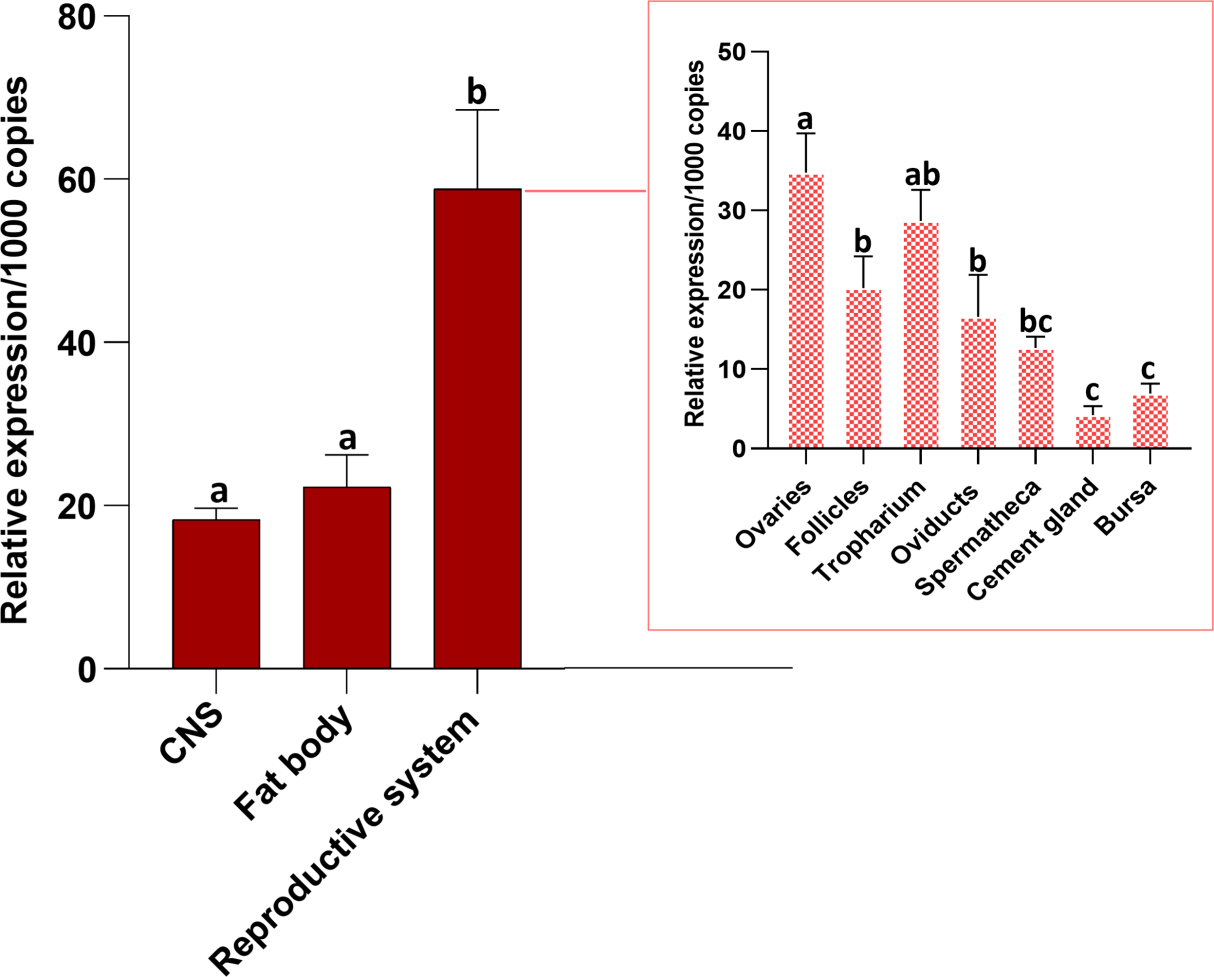
Tissue distribution of *R. prolixus corticotropin-releasing factor/diuretic hormone receptor 2 (Rhopr-CRF/DH-R2)* transcript in the central nervous system (CNS), fat body and reproductive system of unfed adult female *Rhodnius prolixus* at 10-12 days post ecdysis. The outset shows transcript expression of the receptor in the various regions of the reproductive system. The *Rhopr-CRF/DH-R2* transcript level in each tissue was quantified by RT-qPCR and analyzed by the 2^−ΔCt^ method. The y axes represent the relative expression obtained via geometric averaging using *Rp49* and *actin* as reference genes. The results are shown as the mean ± SEM (n = 4-5, where each n represents a pool of tissues from 3 insects). Statistical analysis was performed using a one-way ANOVA and Tukey’s test for multiple comparisons. Significance of P< 0.05 is denoted using letters to indicate bars that are significantly different from others.

Transcript expression of *Rhopr-CRF/DH-R2* (**Figure 2**) was investigated in unfed mated adult females by RT-qPCR. In *R. prolixus* fifth instar, *Rhopr-CRF/DH-R2* transcript is shown to be present in a wide range of peripheral tissues (8), including in the immature female reproductive system. Here, we note higher *Rhopr-CRF/DH-R2* transcript expression levels in the adult female *R. prolixus* reproductive system compared to the CNS and the fat body. Within the reproductive system, highest transcript levels are found in the ovaries, which include the tropharium and follicles, followed by the oviducts, with lower expression in the cement gland and bursa (**Figure 2**).

### 3.3 Temporal transcript expression of *Rhopr-CRF/DH-R2* in the reproductive system of adult females

**Figure 3:**
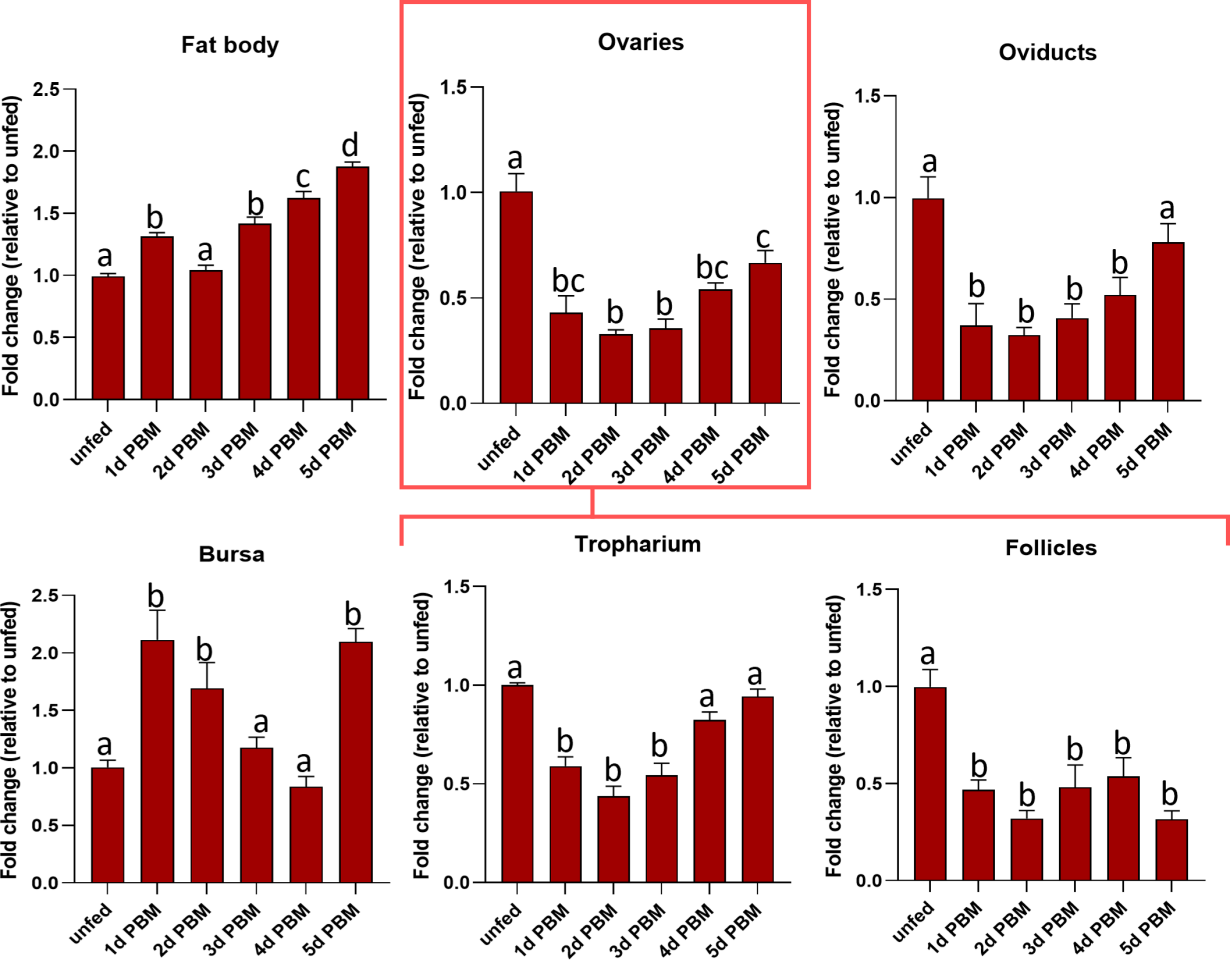
Transcript expression levels of *R. prolixus corticotropin-releasing factor/diuretic hormone receptor 2* (*Rhopr-CRF/DH-R2*) in the fat body and regions of the reproductive system of adult female *R. prolixus*, 10-12 days post ecdysis, before and after feeding. Fat body, ovaries, tropharium, follicles, oviducts, and bursa were analyzed for *Rhopr-CRF/DH-R2* transcript from unfed insects and 1 to 5 days post blood meal (PBM). Transcript expression levels of *Rhopr-CRF/DH-R2* were quantified using RT-qPCR and the 2− ^ΔΔCt^ method. Results are shown as mean ± SEM (n = 4, where each n represents a pool of tissues from 2 insects). Statistical analysis was performed using a one-way ANOVA test with Tukey’s multiple comparisons. Significance of P< 0.05 is denoted using different letters to indicate bars that are significantly different from others.

*Rhopr-CRF/DH-R2* transcript levels were examined in the fat body and reproductive tissues in both unfed and fed adult females (**Figure 3**). The fat body (the main producer of yolk precursor proteins (YPPs), including Vg, needed for egg growth) shows a gradual increase in expression levels of *Rhopr-CRF/DH-R2* as the days post-feeding advance, reaching highest level at 5 days PBM compared to unfed insects. *Rhopr-CRF/DH-R2* in the ovaries, tropharium and oviducts exhibited a drop in expression after feeding followed by a slight increase in expression at 4 and 5 days PBM. *Rhopr-CRF/DH-R2* transcript levels in the follicles dropped after feeding and remained low over the five days. *Rhopr-CRF/DH-R2* expression in the bursa showed an increase in expression after feeding which decreased as the days post-feeding advance with an increase seen on day 5 PBM (**Figure 3**).

### 3.4 Knockdown of *Rhopr-CRF/DH-R2* transcript and effects on numbers of eggs laid and hatching ratio

To investigate the role of Rhopr-CRF/DH signaling in reproductive physiology of *R. prolixus* adult females, *Rhopr-CRF/DH-R2* was downregulated using RNAi. Changes in *Rhopr-CRF/DH-R2* expression levels were verified in the ovaries using RT-qPCR, with a reduction of approximately 75% observed at 2 days following dsCRFR2-injection relative to the dsARG-injected insects and the level remaining reduced until 7 days PBM. However, expression levels recovered by 16-day PBM (**Supplementary Figure 1**).

**Figure 4:**
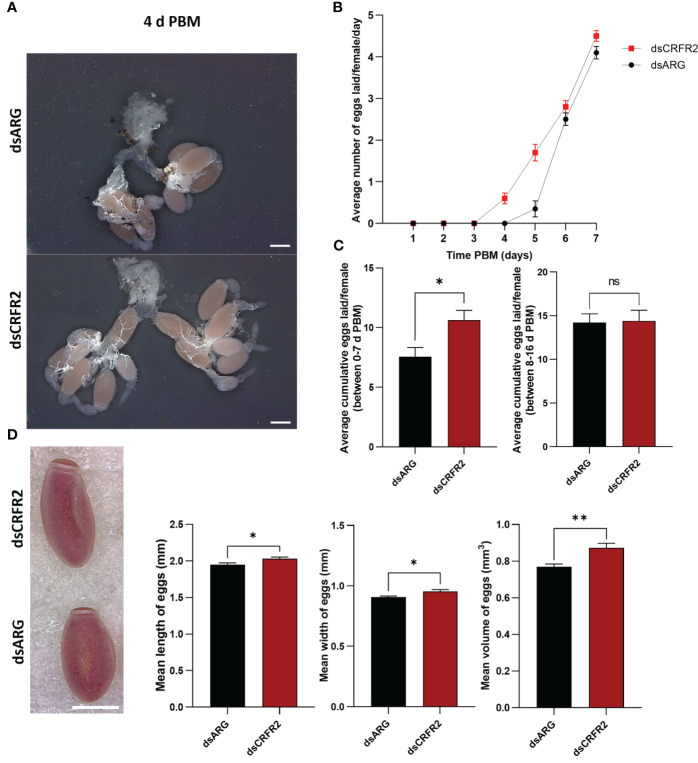
Effects of *corticotropin-releasing factor/diuretic hormone receptor 2 (Rhopr-CRF/DH-R2)* mRNA knockdown on egg laying. **(A)** The reproductive systems from dsARG- and dsCRFR2-injected insects 4 days post blood meal (PBM). Note the chorionated eggs, and their presence in the lateral oviducts in the dsCRFR2-injected insects. Scale bar; 1 mm. **(B)** The average number of eggs laid per female per day over 7 days PBM (mean ± SEM; n= 16). Note that dsCRFR2-injected insects start to lay eggs earlier. **(C)** The cumulative average number of eggs laid per female over 7 days PBM (left histogram) and during 8-16 days PBM (right histogram). Statistically significant differences were determined by Student’s t-test. **(D)** The effects of *Rhopr-CRF/DH-R2* mRNA knockdown on egg phenotype and size of laid eggs from dsCRFR2-injected and dsARG-injected insects (representative images from n =10 eggs per group). Scale bar: 1 mm. Histograms displayed on the right show the measurement of the length, width, and volume of eggs (results are shown as mean ± SEM of n = 10 eggs). Statistically significant differences were determined by Student’s t-test. *p<0.05, **p<0.01, ns, not significant.

Egg production was evaluated in dsARG-injected and dsCRFR2-injected insects by counting the number of eggs laid and their hatching rate. *Rhopr-CRF-R2* knockdown resulted in accelerated egg production, and by 4 d PBM there were already chorionated eggs in the ovaries and lateral oviducts; none in the controls (**Figure 4A**). At 4 d PBM, only the dsCRFR2-injected insects were laying eggs (**Figure 4B**) and by 7 d PBM they had laid more eggs than the dsARG-injected insects (control) (**Figure 4C**). The average number of eggs laid by each female per day for dsCRFR2-injected insects is greater than the controls between feeding and 7 d PBM and similar from 8 d PBM to 16 d PBM (**Figure 4C**) when knockdown has recovered. The length and width of the eggs laid by *Rhopr-CRF/DH-R2* knockdown insects was significantly greater than control insects, as was the volume of the eggs laid (**Figure 4D**).

**Figure 5:**
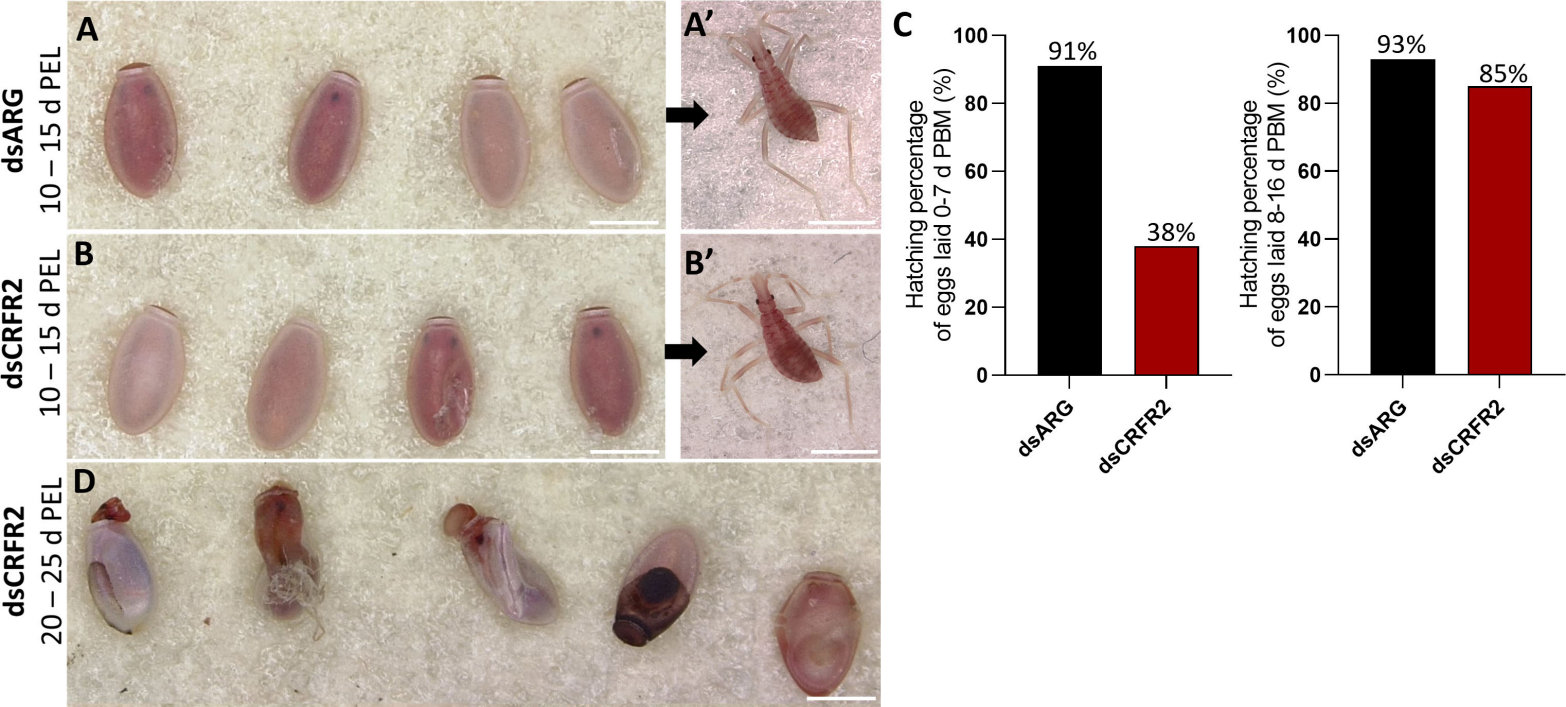
Effect of dsRNA treatment on egg phenotype and hatching. **(A)** Egg phenotype at 10-15 days post egg laying (PEL) for dsARG-injected insects and **(A’)** shows recently hatched insect. Phenotype and first instar insect are representative of n = 10. **(B)** Egg phenotype at 10- 15 days post egg laying from dsCRFR2-injected insects and **(B’)** shows recently hatched insect. Phenotype and first instar insect are representative of n =10. **(C)** Percentage of eggs hatching from dsARG- injected and dsCRFR2-injected insects (305 eggs laid for dsARG-injected insects and 358 for dsCRFR2-injected insects). **(D)** Phenotype of unsuccessful hatching at 20-25 days post egg laying for dsCRF2-injected insects. Scale bars: 1 mm for all images.

Overall, other than the size of the eggs, those laid by dsARG-injected and dsCRFR2-injected insects looked similar, as did the first instars that hatched successfully (**Figures 5A-A’, B-B’**). However, by 10 to 15 days after egg-laying in the dsCRFR2-injected insects only 38% of eggs laid between feeding and 7 days PBM successfully hatched, whereas in the dsARG-injected insects, 91% of the eggs successfully hatched (**Figure 5C**). For eggs laid between 8-16 d PBM there was no statistical difference in hatching rate between the two groups. In addition, of the eggs that did not hatch in the *Rhopr-CRF/DH-R2* knockdown insects, some of them attempted to hatch but were unsuccessful, with some instars trapped in the egg case, others dying in the process of hatching (**Figure 5D**). There was no difference in size between insects that successfully hatched from dsCRFR2-injected females when compared to insects successfully hatched from dsARG-injected females.

### 3.5 *Rhopr-CRF/DH-R2* signaling regulates the production of the yolk precursor protein, Vg, in the fat body and ovaries

**Figure 6:**
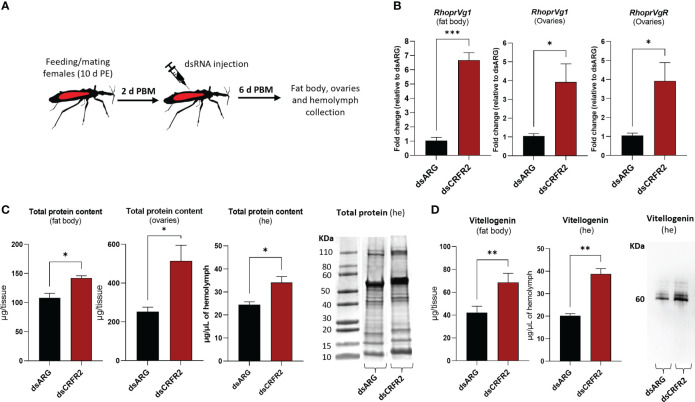
Effects of dsRNA treatment on vitellogenesis at 6 days post blood meal (PBM). **(A)** Experimental scheme. Insects, 10 days post ecdysis (PE), were fed and mated and then injected with dsRNA 2 days PBM. Tissues were collected 4 days later for analysis. **(B)** *RhoprVg1* and *RhoprVgR* mRNA expression in the fat body and ovaries after dsARG- and dsCRFR2-injection. Transcript levels were quantified using RT-qPCR and analyzed by the 2^-^ **^ΔΔCt^** method. The y axes represent the fold change in expression relative to control (dsARG, value ~ 1) obtained via geometric averaging using *Rp49* and *actin* as reference genes. The results are shown as the mean ± SEM (n = 5-6, where each n represents an individual tissue from 1 insect). Statistically significant differences were determined by Student’s t-test. **(C)** Histograms of protein content in the fat body, ovaries and hemolymph (he) of dsRNA-injected females. The results are shown as the mean ± SEM (n = 5, where each n represents the fat body or ovary from 1 insect or n= 4-5 for hemolymph, where each n represents the hemolymph from 1 insect). Statistically significant differences were determined by Student’s t-test. Image of SDS-PAGE analysis of hemolymph (1 μl) after downregulation of *CRFR2* transcript. Image representative of 3 independent experiments. **(D)** Histogram shows quantification of vitellogenin in the fat body and in the hemolymph of dsRNA-injected females by ELISA. The results are shown as the mean ± SEM (n = 4-5, where each n represents the fat body or hemolymph from 1 insect). Statistically significant differences were determined by Student’s t-test. Image of Western blot showing vitellogenin in the hemolymph of dsRNA-injected females (1 μl of 1:20 dilution of hemolymph:carbonate buffer). *p<0.05; **p<0.01; ***p<0.001.

To further examine the involvement of *Rhopr-CRF/DH-R2* signaling in egg production, we measured transcript expression of *R. prolixus Vg* (*RhoprVg1*) in the fat body and ovaries, as well as the *Vg* receptor (*RhoprVgR*) transcript in the ovaries following *Rhopr-CRF/DH-R2* knockdown (**Figure 6**). *RhoprVg1* transcript levels in the fat body and ovaries are significantly increased, in addition to an increase in *RhoprVgR* transcript levels in the ovaries at 6 d PBM in dsCRFR2-injected insects (**Figure 6B**). The total protein content in the fat body, ovaries and in the hemolymph of dsCRFR2-injected insects is greater than in the controls, and these differences are verified for the hemolymph on Coomassie blue stained gels (**Figure 6C**). The changes in total protein content are due in part to the increase in the main YPP, Vg, in the fat body and hemolymph, as determined by ELISA and Western blot (**Figure 6D**).

**Figure 7:**
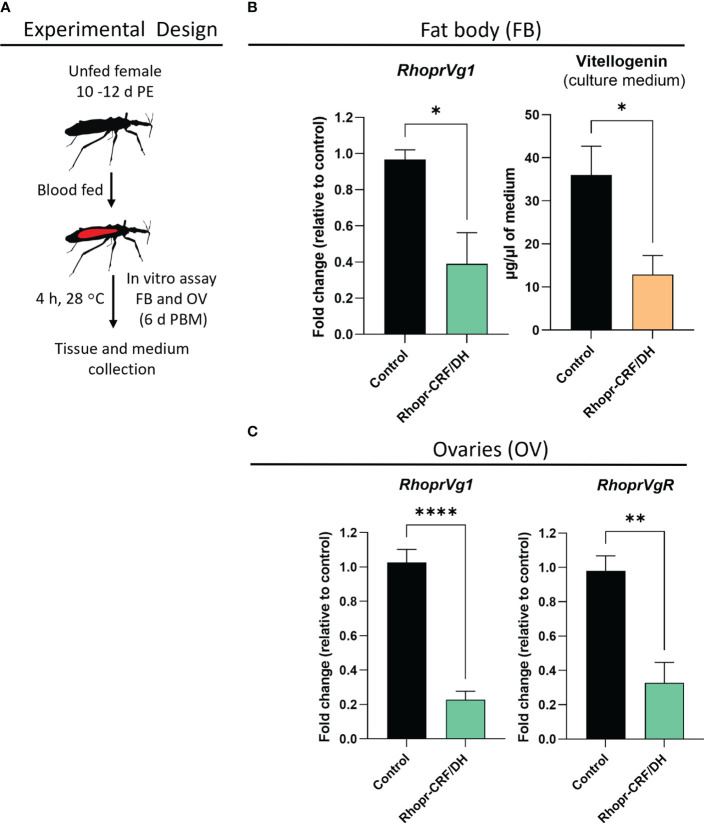
Effects of *Rhodnius prolixus* corticotropin-releasing factor/diuretic hormone (Rhopr-CRF/DH) on vitellogenin (Vg) production *in vitro*. **(A)** Experimental design for *in vitro* assay of fat body (FB) and ovaries (OV) from fed females (10-12 days post ecdysis, PE) at 6 days post blood meal (PBM). **(B)** Rhopr-CRF/DH (10^-8^ M) or the same volume of saline (control) were added to the culture medium and four hours later *RhoprVg1* transcript levels quantified in the fat body and vitellogenin titer quantified in the incubation medium using ELISA, and in **(C)** *RhoprVg1* and *RhoprVgR* transcript levels quantified in the ovaries. Transcript levels were quantified using RT-qPCR and analyzed by the 2^− ΔCt^ method. The y axes represent the fold change in expression relative to control (saline incubation, black column, value ~1) obtained via geometric averaging using *Rp49* and *actin* as reference genes. The results are shown as the mean ± SEM (n = 4, where each n represents fat body or ovary from 1 insect). Statistically significant differences were determined by Student’s t-test. *p<0.05; **p<0.01; ****p<0.0001.

**Figure 8:**
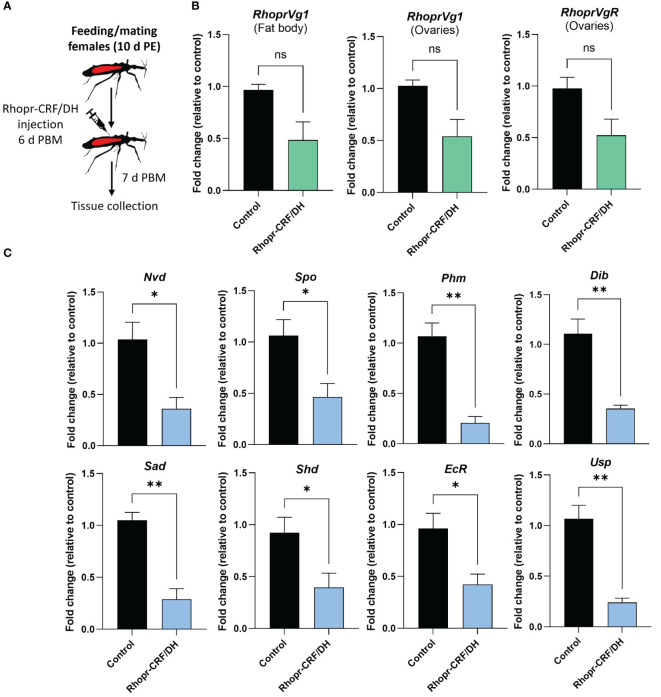
Effects of *Rhodnius prolixus* corticotropin-releasing factor/diuretic hormone (Rhopr-CRF/DH) on ecdysteroid production *in vivo*. **(A)** Experimental design. Adult females 10 d post ecdysis (PE) were fed and allowed to mate, and at 6 days post blood meal (PBM) insects were injected with Rhopr-CRF/DH (2 uL of 10^-3^ M) and 24 hours later the ovaries were dissected to measure **(B)** transcript levels of *RhoprVg1* in the fat body and ovaries, *RhoprVgR* in ovaries and **(C)** biosynthetic enzymes and ecdysteroid receptor subunits were quantified using RT-qPCR and analyzed by the 2^− ΔCt^ method. The y axes represent the fold change in expression relative to control (saline injection, black column, value ~1) obtained via geometric averaging using *Rp49* and *actin* as reference genes. The results are shown as the mean ± SEM (n = 4, where each n represents an ovary from 1 insect). Statistically significant differences were determined by Student’s t-test. *p<0.05; **p<0.01; not significant, ns. Neverland (Nvd), Spook (Spo), Phantom (Phm), Disembodied (Dib), Shadow (Sad), Shade (Shd), Ecdysone Receptor (EcR) and Utraspiracle (USP).

We also performed *in vitro* assays to examine the direct effect of Rhopr-CRF/DH on the production of Vg in the fat body and ovaries of adult female *R. prolixus* (**Figure 7**). Interestingly, *RhoprVg1* transcript levels are notably reduced in the fat body, with a significant reduction in Vg titer in the fat body culture medium, as determined by ELISA (**Figure 7B**). *RhoprVg1* and *RhoprVgR* expression levels are remarkably reduced in ovaries (**Figure 7C**). An *in vivo* assay examining the effect of injecting Rhopr-CRF/DH on transcript expression of *RhoprVg1* in the fat body and ovaries and *RhoprVgR* in the ovaries exhibited a similar trend of decreased transcript levels in insects injected with Rhopr-CRF/DH compared to the control insects (**Figure 8A, B**).

### 3.6 *Rhopr-CRF/DH-R2* signaling regulates transcript expression of ecdysteroid biosynthetic enzymes, and ecdysone receptor complex

**Figure 9:**
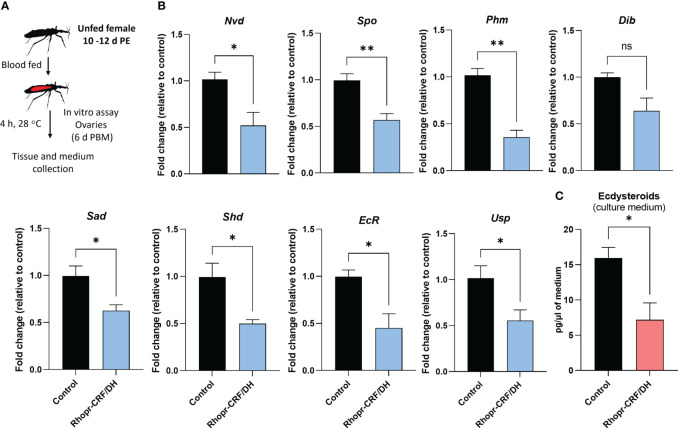
Effects of *Rhodnius prolixus* corticotropin-releasing factor/diuretic hormone (Rhopr-CRF/DH) on ecdysteroid production in the ovary *in vitro*. **(A)** Experimental design. Ovaries from fed females (10-12 days post ecdysis, PE) at 6 days post blood meal (PBM) were incubated for four hours in culture medium containing Rhopr-CRF/DH (10^-8^ M) or the same volume of saline (control). **(B)** Transcript levels of the biosynthetic enzymes and receptor subunits were quantified using RT-qPCR and analyzed by the 2^− ΔCt^ method. The y axes represent the fold change in expression relative to control (black column, value ~1) obtained via geometric averaging using *Rp49* and *actin* as reference genes. The results are shown as the mean ± SEM (n = 4, where each n represents an ovary from 1 insect). Significance determined by Student’s t-test. **(C)** Ecdysteroid titers in the incubation medium were quantified by ELISA. The y axes represent ecdysteroid levels of the medium. The results are shown as the mean ± SEM (n = 4, where each n represents the incubation medium that contained the ovary from 1 insect). Statistically significant differences were determined by Student’s t-test. *p<0.05; **p<0.01; ns, not significant; Nvd, Neverland; Spo, Spook; Phm, Phantom; Dib, Disembodied; Sad, Shadow; Shd, Shade; EcR, Ecdysone Receptor; USP, Utraspiracle.

Given that ecdysteroids are made by growing follicles, we quantified transcript expression levels of the biosynthetic enzymes for ecdysteroids, including Halloween genes, neverland and the ecdysone receptor complex, in the ovaries, via *in vivo* and *in vitro* assays using Rhopr-CRF/DH (**Figures 8C**, **9A, B**). Transcript expression levels of Halloween genes and the ecdysone receptor complex were significantly decreased in the ovaries of insects injected with Rhopr-CRF/DH and in ovaries incubated for 4 hours with Rhopr-CRF/DH (**Figures 8C**, **9B**). In addition, downregulation of *Rhopr-CRF/DH-R2* mRNA using RNAi led to an increase in transcript expression levels of *Shadow* (*Sad*) and *Shade* (*Shd*) in the ovaries, 6 d PBM (**Supplementary Figure 3**); an effect that is opposite to that seen with application of Rhopr-CRF/DH.

To verify whether *Rhopr-CRF/DH-R2* signaling interferes with egg development due to a reduced ecdysteroid signaling, we quantified ecdysteroid in culture medium containing ovaries. Interestingly, ecdysteroid levels were remarkably reduced to approximately 40% compared to controls (**Figure 9C**).

## 4 Discussion

Neuropeptides play a significant role in the regulation of many physiological processes, including reproduction in insects. Insect reproduction is a complex and tightly regulated process, and neuropeptides serve as important signaling molecules that orchestrate and modulate different aspects of this vital biological function. In this study, we focus on the role of Rhopr-CRF/DH signaling in reproduction by examining the effects of *Rhopr-CRF/DH-R2* downregulation on oogenesis and egg production in adult female *R. prolixus.*

The distribution pattern of neurons displaying CRF-like immunoreactivity in the CNS of *R. prolixus* has previously been described in great detail (14, 31). In particular, CRF-like immunoreactivity is found in 5 bilaterally paired NSCs in the MTGM, with processes extending to the abdominal neurohemal sites on abdominal nerves 1, 2 and 3. It has been shown that these cells release Rhopr-CRF/DH as a diuretic hormone (see reference 20). In a more recent study, we reported GPB5-like immunoreactive neurons throughout the CNS, and found a similar distribution pattern to that of CRF/DH-like immunoreactivity (21). Here, we use double-label immunohistochemistry to show the co-localization of CRF/DH-like immunoreactivity and GPB5-like immunoreactivity in the lateral NSCs in the MTGM and their neurohemal sites on the abdominal nerves, suggesting these peptides might be released together to regulate important processes associated with feeding, diuresis and reproduction.

The glycoprotein hormone GPA2/GPB5 plays a key role in reproduction in adult female *R. prolixus*, acting as gonad-inhibiting hormone (22); and disruption of the GPA2/GPB5 signaling pathway by RNAi leads to accelerated oogenesis, an increase in the number of eggs produced and laid, an increase in egg size and a reduction in hatching rate. We therefore conjectured that Rhopr-CRF/DH may perform a similar function, since in locusts, injection of CRF/DH into mature *S. gregaria* females results in significantly smaller oocytes, and in reduced ecdysteroid levels circulating in the hemolymph (9). Similarly, injections of Rhopr-CRF/DH into female *R. prolixus* leads to a reduced number of eggs produced (14). We therefore investigated the role of Rhopr-CRF/DH in reproduction in *R. prolixus* in more detail.

The transcript expression of *Rhopr-CRF/DH-R2* in the fat body and reproductive tissues of adult female *R. prolixus* are elevated PBM, further suggesting the involvement of the Rhopr-CRF/DH signaling pathway in oocyte development and in regulating the production of Vg by the fat body. In *R. prolixus*, YPPs are primarily synthesized by the fat body and these YPPs, including the main YPP, Vg, are taken up by growing oocytes. Indeed, knocking down *Rhopr-CRF/DH-R2* results in an increase in *RhoprVg1* transcript expression in the fat body and ovaries, in addition to an increased *RhoprVgR* expression in the ovaries. dsCRFR2 consequentially results in an accelerated egg maturation and an increase in the number of eggs laid after feeding compared to the controls. Knockdown also results in larger eggs and an increase in the cumulative number of eggs produced. The outcomes of dsCRFR2 on egg production is remarkably similar to that previously reported for downregulation of *LGR1*, the receptor for GPA2/GPB5 (22). An effect on nutrient production is also evident in dsCRFR2-treated insects, which show an accumulation of total protein in the fat body and in the hemolymph, with Vg as a major contributor. This increase in hemolymph Vg, together with the increased *RhoprVgR* transcript expression in the ovaries, would enable a greater uptake into the oocytes of Vg from the hemolymph. Interestingly, even though the ovaries are not the main source of Vg production, the results suggest that *Rhopr-CRF/DH* signaling regulates the amount of Vg produced by the ovaries. The egg size in dsCRFR2 treated *R. prolixus* is significantly greater than in the controls, suggesting that downregulation of *Rhopr-CRF/DH-R2* signaling accelerates and increases the amount of YPPs produced by the fat body and taken into the developing eggs. Since the downregulation of *Rhopr-CRF/DH-R2* transcript is transient and eliminated by 16 d PBM, the effect of *Rhopr-CRF/DH-R2* knockdown cannot be evaluated beyond 7 d PBM; but the effect might mirror that of dsLGR1 (22) if the transcript were to remain reduced, as it is for *LGR1*. It is worth noting that a previous study showed that injection of mature *S. gregaria* females with Schgr-CRF/DH resulted in the production of significantly smaller oocytes, and in reduced levels of ecdysteroids circulating in the haemolymph (9). Knockdown of the Schgr-CRF/DH transcript reversed the effects on reproduction.

A previous study illustrated the importance of ecdysteroid for egg production in *R. prolixus* and knocking down the ecdysone receptor and shade reduces the number of eggs made and laid, and influences the maturation of oocytes (30). In these experiments, there is also a significant reduction in *Rhopr Vg1* transcript levels in the fat body, suggesting the ecdysteroid signaling pathway is involved with Vg synthesis in the fat body (30). Here, *in vitro* and *in vivo* experiments revealed that Rhopr-CRF/DH signaling significantly reduces expression of the Halloween gene transcripts (*spo*, *phm*, *dib*, *sad* and *shd*), the non-Halloween gene transcript (*nvd*), as well as the genes encoding the ecdysteroid receptor, EcR and USP in the ovaries, indicating that there might be a decrease in ecdysteroid titer in the hemolymph. Indeed, the application of Rhopr-CRF/DH to the isolated ovary decreases the levels of ecdysteroids appearing in the medium by approximately 40%, supporting the role of Rhopr-CRF/DH signaling in regulating ecdysteroids titers.

In Blattodea and Hemiptera, including *R. prolixus*, juvenile hormone (JH) is essential for full egg production as it regulates the production of Vg in the fat body as well as its uptake by the ovary in *R. prolixus* (32). In *D. melanogaster*, Vg synthesis in the fat body and uptake by the oocytes is controlled by ecdysteroid levels (33, 34). It is suggested there may be crosstalk between JH and ecdysteroids in regulating Vg in the fat body (30). Therefore, it is worth mentioning that the effect seen by Rhopr-CRF/DH treatment *in vivo* on Vg levels might be influenced by an increased JH titer PBM, although the *in vitro* assay also indicates that the effects of Rhopr-CRF/DH on Vg expression can be direct on the fat body.

Downregulating *Rhopr-CRF/DH-R2* in the adult female *R. prolixus* also leads to a lower hatching rate and unsuccessful hatching in a significant number of eggs. Insects that died during hatching tended to be unable to pass successfully through the operculum, indicating a possible disruption of the normal hatching behavior; an interesting observation since CRF/DH has been proposed to play a part in ecdysis behavior (15). Also, although the size of eggs from the dsCRFR2-treated and control insects were different, those that successfully hatched in the dsCRFR2-treated insects had similar body morphology and body length to the control insects.

In conclusion, these results, coupled with the earlier data of Al-Dailami et al. (22) and Mollayeva et al (14), suggest that in adult female *R. prolixus*, the CRF/DH and GPA2/GPB5 signaling pathways which are activated by a blood meal might delay egg production, possibly until nutrients from the blood meal are fully available for vitellogenesis, and/or in a virgin, possibly delaying until mating occurs. They may act as a stop/go signal in light of the very energy demanding process of reproduction. Disruption of these pathways by RNAi leads to accelerated oogenesis, an increase in the number of eggs produced and laid, an increase in egg size and a reduction in hatching rate. The colocalization of these peptides in the CNS suggests that Rhopr-CRF/DH might be working in conjunction with the GPA2/GPB5 to regulate vital processes in the insect, such as feeding and diuresis; and in reproduction, by acting as gonad-inhibiting hormones.

## Data availability statement

The original contributions presented in the study are included in the article and **Supplementary Material**. Further inquiries can be directed to the corresponding author.

## Ethics statement

The manuscript presents research on animals that do not require ethical approval for their study.

## Author contributions

AA-D: Writing – original draft, Conceptualization, Data curation, Formal Analysis, Investigation, Methodology, Validation, Visualization. IO: Conceptualization, Formal Analysis, Funding acquisition, Investigation, Project administration, Resources, Supervision, Validation, Visualization, Writing – review & editing, Methodology. AL: Conceptualization, Formal Analysis, Funding acquisition, Investigation, Project administration, Resources, Supervision, Validation, Visualization, Writing – review & editing, Methodology.
