# Supplementary material for: Corticotropin-releasing factor-like diuretic hormone acts as a gonad-inhibiting hormone in adult female, Rhodnius prolixus
Source: Front Endocrinol (Lausanne). 2023 Sep 29;14:1279929. doi: 10.3389/fendo.2023.1279929 (PMC10570869; doi:10.3389/fendo.2023.1279929)
Supplement: Supplementary file 1 [file DataSheet_1.pdf]

Suppl. Table 1. Gene-specific primers used for qPCR and dsRNA experiments.

**Oligo Name**	**Oligo Sequence 5'- 3'**
CRFR2_F	ACTGCTCTTGGTTTGGCAGT
CRFR2_R	GCCGGGTCTGTTTTAACGTA
Vg1_F	TTGCTAGTCGCATGAACCTG
Vg1_R	TTTAGTGGTGCATCGCTCTG
VgR_F	GTGAAACTCAGGAGAAATTGGC
VgR_R	AGGACACACCATGCGCTATC
Rp49_F	ACCAATGGAAGTAACCGCCT
Rp49_R	AGGACACACCATGCGCTATC
Actin_F	AGAGAAAAGATGACGCAGATAATGT
Actin_R	ATATCCCTAACAATTTCACGTTCG
CRFR2 i_F	ACTGCTCTTGGTTTGGCAGT
CRFR2 i_R	GCCGGGTCTGTTTTAACGTA
CRFR2 ii_F	TGGGCATCACGAAGGTTGTA
CRFR2 ii_R	ATGTGTAGCGTCTTGCTCCC
ARG_F	ATGAGTATTCAACATTTCCGTGTC
ARG_R	AATAGTTTGCGCAACGTTG
CRFR2 i_F_T7	**TAATACGACTCACTATAGGGAGA**ACTGCTCTTGGTTTGGCAGT
CRFR2i_R_T7	**TAATACGACTCACTATAGGGAGA**GCCGGGTCTGTTTTAACGTA
CRFR2 ii_F_T7	**TAATACGACTCACTATAGGGAGA**TGGGCATCACGAAGGTTGTA
CRFR2 ii_R_T7	**TAATACGACTCACTATAGGGAGA** ATGTGTAGCGTCTTGCTCCC
ARG F_T7	**TAATACGACTCACTATAGGGAGA**ATGAGTATTCAACATTTCCGTGTC
ARG R_T7	**TAATACGACTCACTATAGGGAGA**AATAGTTTGCGCAACGTTG

* **TAATACGACTCACTATAGGGAGA** = T7 RNA polymerase promoter region
