# Supplementary material for: Corticotropin-releasing factor-like diuretic hormone acts as a gonad-inhibiting hormone in adult female, Rhodnius prolixus
Source: Front Endocrinol (Lausanne). 2023 Sep 29;14:1279929. doi: 10.3389/fendo.2023.1279929 (PMC10570869; doi:10.3389/fendo.2023.1279929)
Supplement: Supplementary file 2 [file DataSheet_2.pdf]

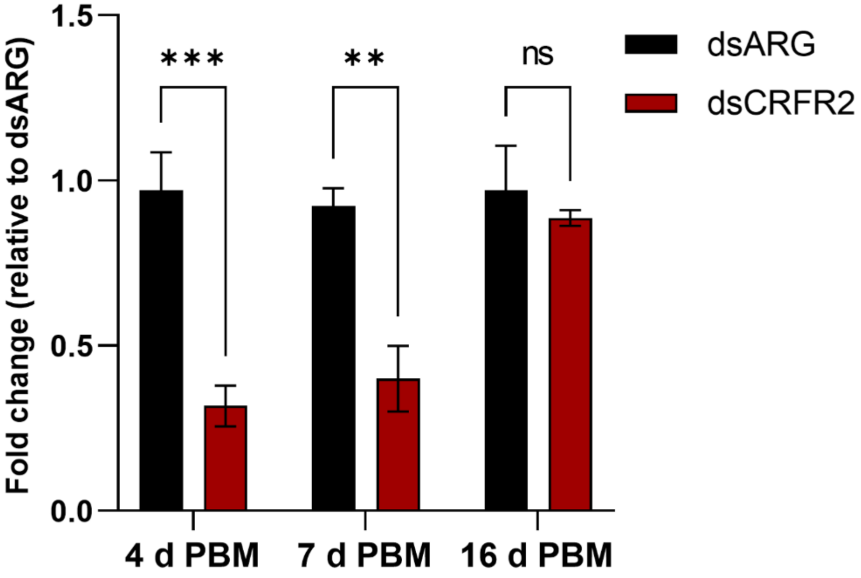

Supplementary Figure 1. Verification of dsRNA efficiency by real time qPCR (n=4). Transcript levels of *Rhodnius prolixus* corticotropin-releasing factor/diuretic hormone receptor 2 (Rhopr-CRF/DH-R2) in the ovaries of fed insects at 4 days (d) post blood meal (PBM) and 7 d PBM are reduced compared to the controls (dsARG-injected). At 16 d PBM, transcript expression levels of Rhopr-CRF/DH-R2 recover to the levels of the control group. Statistically significant differences were determined by Student's t-test. ns, not significant, **$p < 0.01$, ***$p < 0.001$.

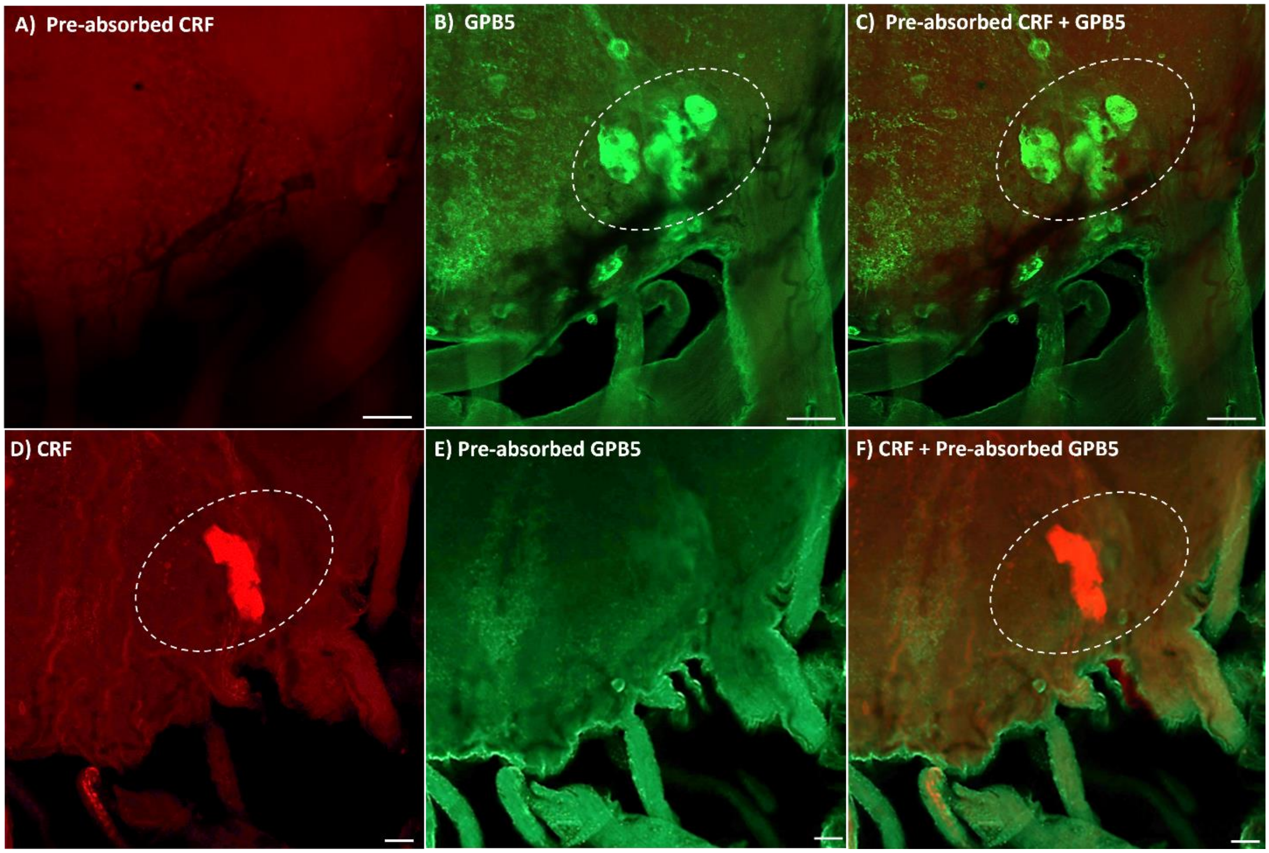

Supplementary Figure 2. Controls for double-label immunohistochemistry of *Rhodnius prolixus* neurosecretory cells in the mesothoracic ganglionic mass. (A–C) representative images showing that preabsorbing the CRF antibody with Rhopr-CRF/DH (10−5 M) abolished CRF-like staining. (D–F) representative images showing that preabsorbing the GBP5 antibody with GPB5 antigen (10−5 M) abolished GPB5-like staining. Scale bars; 50 mM

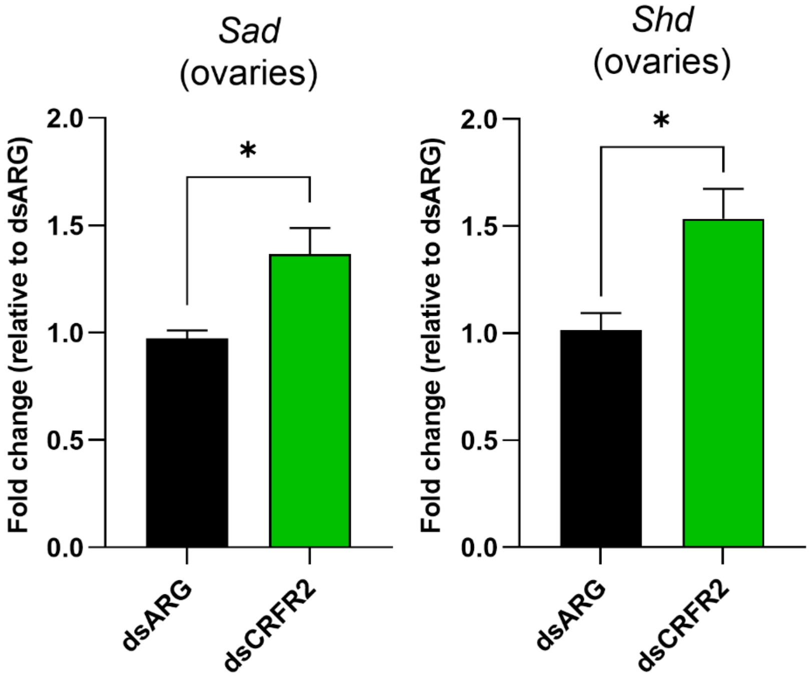

Supplementary Figure 3. Verification of dsRNA efficiency by real time qPCR (n=4). Transcript levels of *Rhodnius prolixus* corticotropin-releasing factor/diuretic hormone receptor 2 (Rhopr-CRF/DH-R2) in the ovaries of fed insects at 4 days (d) post blood meal (PBM) and 7 d PBM are reduced compared to the controls (dsARG-injected). At 16 d PBM, transcript expression levels of Rhopr-CRF/DH-R2 recover to the levels of the control group. Statistically significant differences were determined by Student's t-test. **$p < 0.01$, ***$p < 0.001$.
